# Biopolymeric Membrane Enriched with Chitosan and Silver for Metallic Ions Removal

**DOI:** 10.3390/polym12081792

**Published:** 2020-08-10

**Authors:** Simona Căprărescu, Roxana Gabriela Zgârian, Graţiela Teodora Tihan, Violeta Purcar, Eugenia Eftimie Totu, Cristina Modrogan, Anita-Laura Chiriac, Cristian Andi Nicolae

**Affiliations:** 1 Department of Inorganic Chemistry, Physical Chemistry and Electrochemistry, Faculty of Applied Chemistry and Materials Science, University POLITEHNICA of Bucharest, Polizu Street No. 1-7, 011061 Bucharest, Romania; scaprarescu@yahoo.com; 2 Department of General Chemistry, Faculty of Applied Chemistry and Materials Science, University POLITEHNICA of Bucharest, Polizu Street No. 1-7, 011061 Bucharest, Romania; roxana.zgarian@upb.ro; 3 The National Institute for Research & Development in Chemistry and Petrochemistry—ICECHIM, Splaiul Independentei No. 202, 060021 Bucharest, Romania; purcarvioleta@gmail.com (V.P.); raduanita@gmail.com (A.-L.C.); ca_nicolae@yahoo.com (C.A.N.); 4 Analytical Chemistry and Environmental Engineering Department, Faculty of Applied Chemistry and Materials Science, University POLITEHNICA of Bucharest, Polizu Street No. 1-7, 011061 Bucharest, Romania; eugenia.totu@upb.ro (E.E.T.); c_modrogan@yahoo.com (C.M.)

**Keywords:** chitosan, silver ions, biopolymers, membrane, electrodialysis, iron ions

## Abstract

The present paper synthesized, characterized, and evaluated the performance of the novel biopolymeric membrane enriched with cellulose acetate and chitosan (CHI)-silver (Ag) ions in order to remove iron ion from the synthetic wastewater using a new electrodialysis system. The prepared membranes were characterized by Fourier Transforms Infrared Spectroscopy-Attenuated Total Reflection (FTIR-ATR), Thermal Gravimetric Analysis (TGA) and Differential Thermal Analysis (DSC), contact angle measurements, microscopy studies, and electrochemical impedance spectroscopy (EIS). The electrodialysis experiments were performed at the different applied voltages (5, 10, and 15 V) for one hour, at room temperature. The treatment rate (T_E_) of iron ions, current efficiency (I_E_), and energy consumption (W_c_) were calculated. FTIR-ATR spectra evidenced that incorporation of CHI-Ag ions into the polymer mixture led to a polymer-metal ion complex formation within the membrane. The TGA-DSC analysis for the obtained biopolymeric membranes showed excellent thermal stability (>350 °C). The contact angle measurements demonstrated the hydrophobic character of the polymeric membrane and a decrease of it by CHI-Ag adding. The EIS results indicated that the silver ions induced a higher ionic electrical conductivity. The highest value of the iron ions treatment rate (>60%) was obtained for the biopolymeric membrane with CHI-Ag ions at applied voltage of 15 V.

## 1. Introduction

The pollution of water with different metallic ions (i.e., Fe^2+^, Cu^2+^, Pb^2+^, Cr^3+^, Ni^2+^, Cd^2+^, Zn^2+^, Co^2+^, Mn^2+^, Pd^2+^), even in trace quantities, becomes a pressing problem for public health and environmental because these are toxic, nonbiodegradable, and damaging to the environment. Wastewater effluents that contain significant quantities of iron ions, present as Fe^2+^ or Fe^3+^, can be provided from many industries, such as automobiles, machinery, tools, paints and treated timber, industrial and domestic equipment, along with smelting, mining industry, metallurgy, clinical activities, erosion, or other geological processes [1,2,3,4].

Iron (Fe) is an essential element necessary for all living organisms in limited amounts. For instance, 70% from three to four grams of iron, which is the whole amount in a body, is under the complex form in red blood cells transporting oxygen to the cells. Also, iron is involved in brain functions or DNA synthesis, and it is bound to transferrin. Although essential for organisms, an excess of Fe^2+^ could transform it into a toxic compound. The iron overdose could be accumulated in the liver, the heart, or in the pancreas, finally damaging them. The iron overdose might appear either due to hemochromatosis (a genetic disorder) or if a healthy person consumes drinking water with Fe^2+^ contents exceeding 200 mg, being lethal within the range of 10–50 g. Such behavior could be ascribed to the specific pro-oxidant action of the uncomplexed or partially complexed Fe^2+^ that could participate in the specific Fenton processes generating reactive oxygen species (ROS) able to damage the cells [5]. Therefore, for the drinking water, there were imposed strict concentration limits to 0.3 mg/L, based on the recommendation of the World Health Organization and by the Environmental Protection Agency [1,2,4].

In the latest years, numerous research studies were focused on the synthesis and preparation of biodegradable and biocompatible materials in order to be used for the removal of various heavy metals from water solutions. Different treatment methods have been successfully applied to remove and recover various heavy metals from different wastewater, such as chemical precipitation, ion exchange, flotation, adsorption, filtration, oxidation-coagulation, membrane technology (e.g., electrodialysis, nanofiltration, reverse osmosis, microfiltration, ultrafiltration). Chemical precipitation, evaporation, solvent extraction, coagulation/flocculation, adsorption, ion exchange are less effective to remove heavy metals (e.g., Cu^2+^, Hg^2+^, Cd^2+^, Zn^2+^) because they require chemicals, energy consumption, and high capital and operational costs. Most of these methods generate the toxic residual metal sludge that must be treated because they affect the environmental if they are eliminated in the water [6]. The electrochemical methods involve the use of a membrane, most often prepared from the polymers, due to their chemical and physical properties [7,8,9,10,11,12,13,14,15,16,17]. The electrochemical methods have been successfully used at laboratory and industrial scale due to the many advantages, such as their technical maturity, low maintenance costs, feasibility, the ability to remove or recover metal ions, to remove organic or inorganic colloids, bacteria or other microorganisms, and lower energy consumption [10,11,12].

Electrodialysis (ED) is an electrochemical membrane process based on bipolar membranes or hybrid membranes, where ions are transported through ion selective membranes from one solution to another under the electric field influence [13,14,15,16,17]. The ED process was successfully applied for the removal of heavy metals from various wastewaters due to the high efficiency or operational feasibility. Also, the process can simultaneously separate, enrich, and concentrate the investigated solutions. This membrane process operates without noise, does not require specialized equipment, has low chemical consumption, and the operation and maintenance are easy [13,14,15,16,17]. Sue et al. [15] studied the efficiency of electrodialysis (ED) process to treat and reclaim wastewater that contains nano-scale slurry particles and metal residues from polishing a wafer. The results indicated that the ED process for removal of Cu^2+^ was more than 99.3% with applied field strength of 1.5 V/cm and reaction time of 3 h. In our previously studies we have successfully applied the electrodialysis in conjunction with copolymers/natural extract membrane for the removal of nickel ions from synthetic wastewater. The results showed that the removal efficiency was higher than 67% for membrane with natural extract, after the 90 min of treatment [13].

The membranes based on natural biopolymers such as chitosan, cellulose, and its derivate were used in many applications (microfiltration, ultrafiltration, reverse osmosis, and nanofiltration membrane processes) due to the their advantages, such as low cost, availability, biodegradability, natural sources, being environmentally friendly, high flux and high rejection, good adsorption of liquids [12,18,19,20,21,22,23,24,25]. Chitosan (CS), due to its advantages such as nontoxicity, being cheap, biocompatibility, and biodegradability [17,18,19,20,21,22,23] has been widely reported as an effective sorbent of heavy metals (e.g., Fe^2+^, Cu^2+^, Pb^2+^, Cr^3+^, Ni^2+^, Cd^2+^, Zn^2+^, Co^2+^, Mn^2+^, and Pd^2+^) from aqueous systems. The ecological chitosan nanofibers produced by electrospinning technique has been successfully applied in the removal of Fe^3+^, Cd^2+^, and Cu^2+^ from industrial effluents [5,7,10,18,26,27]. The chitosan-based biopolymers have a good capacity for metal ions adsorption, including Cu^2+^, Zn^2+^, Pb^2+^, Ni^2+^, and Cd^2+^, due to the amino group presence, which is responsible for metal ion binding by chelation mechanism [26,27,28,29,30,31]. Furthermore, the inclusion of silver ions into the polymeric matrix has the advantage of improving the antimicrobial effect while maintaining low cytotoxicity. The development of polymer membranes based on chitosan containing silver nanoparticles has been applied in order to remove the different heavy metals (e.g., Fe^2+^, Hg^2+^, Pb^2+^, Cu^2+^, Cd^2+^, Ni^2+^, and Cr^3+^), for various environmental remediation and water purification applications [30,31,32,33,34].

Reiad et al. [35] studied the adsorptive removal of iron and manganese ions from aqueous solutions by microporous chitosan (CS)/polyethylene glycol (PEG) blend membrane. The prepared membranes, investigated on batch adsorption, presented the adsorption capacities as follows: up to 38 mg/g for Fe^2+^ at pH = 5, within 60 min, and up to 18 mg/g for Mn^2+^ at pH = 5.9, within 65 min. Xiong X et al. [36] prepared a cellulose/chitosan hybrid membrane with chelating metal properties by regenerating dissolved cellulose in an acetic acid solution containing chitosan. The hybrid membrane was applied for nanofiltration and complexation of Cu^2+^ with amino groups. The studies indicated that the rejection was 90.1% when the pH of the feed solution was five. Wang et al. [37] reported the preparation of composite membrane with a SiO_2_ microsphere-free porous surface and inner pores with immobilized SiO_2_ microspheres decorated by silver nanoparticles. Zdorovets et al. [38] studied the modification of flexible track-etched membranes as the basis for the sensor with various polymers and their influence on the accuracy of copper, cadmium, and lead ions detection in water. They demonstrated that the modification of membranes by copolymers with carboxylic and amino groups leads to more accurate detection of heavy metal ions. A review presented by Choi et al. [39] showed the advantages of polymer membrane used at a high temperature (100~200 °C). The phosphoric-acid-containing polymeric material synthesized from cross-linked polybenzoxazine presented feasible characteristics.

At present, there have not been any reported studies about the removal of iron ions from wastewater using electrodialysis process in combination with biopolymer membranes with silver particles content. It is important to understand the performance of membranes and electrodialysis system for the removal of iron ions from wastewater in laboratory or pilot plant conditions. Therefore, the present study refers to the synthesis, characterization, and efficiency of the novel biopolymeric membrane containing cellulose acetate (CA) and polyethylene glycol (PEG), with and without chitosan (CHI) and silver (Ag) ions for the iron ion removal from synthetic wastewater applying the electrodialysis process. The chemical structure, wettability, morphological aspect, and electrochemical behavior were evaluated by Fourier Transforms Infrared Spectroscopy-Attenuated Total Reflection (FTIR-ATR), contact angle measurements, surface free energy (SFE), microscopy studies, TGA-DSC analysis, and electrochemical impedance spectroscopy. Properties of obtained biopolymeric membranes were correlated with their ability to retain iron ions from synthetic wastewater. The influence of applied voltage on the electrodialysis system, using the prepared synthetic industrial wastewater, quality of permeates samples, and performances of the biopolymeric membranes were investigated.

## 2. Materials and Methods

### 2.1. Materials

All chemical reagents were analytical grade and used without further purification. White cellulose acetate (CA) powder (average M_n_ ~30,000, density 1.3 g/mL, purity 99%), silver nitrate (AgNO_3_, 99.0%), ferrous sulfate heptahydrate (FeSO_4_·7H_2_O), acetic acid, and polyethylene glycol 200 (PEG) (density 1.124 g/cm^3^ at 20 °C, molar mass 190–210 g/mol) were purchased from Sigma-Aldrich (Merck KGaA, Darmstadt, Germany). Chitosan (CHI) (powder, low-viscous, viscosity <200 mPa.s; deacetylation degree ≥75%) was purchased from Sigma-Aldrich (Merck KGaA, Darmstadt, Germany). Sulfuric acid was supplied by Merck (Merck KGaA, Darmstadt, Germany). The synthetic wastewater solutions were prepared with distilled water.

### 2.2. Biopolymeric Membrane Preparation

The biopolymeric membranes were obtained by wet-phase inversion method at room temperature. Firstly, the chitosan-based AgNO_3_ solution (CHI-Ag) was prepared as follows: a quantity of chitosan powder (1 g) was dissolved in the acetic acid solution (1%). The solution was heated for 1 h at 60 °C and magnetically stirred (500 rpm) until the chitosan was dissolved entirely. After that, 20 mL of AgNO_3_ (0.1 N) was added over chitosan solution and magnetically stirred (500 rpm) at 60 °C for 3 h. The solution was then left overnight to cool at room temperature (22 ± 1 °C).

The casting solution and biopolymeric membrane were prepared as follows: a mixture of 4 g cellulose acetate, 0.5 mL CHI-Ag, and 0.3% (wt.%) PEG was completely dissolved in 44 mL acetic acid (8 wt.% polymer solution) at 75 °C under magnetic stirring (200 rpm) for about 4 h. The obtained homogeneous solution was kept at room temperature (22 ± 1 °C) without stirring for one day to remove the formed oxygen bubbles. After that, the solution was spread uniformly on the glass plate surface with a thickness of about 1 mm, using a special knife. The cast film, together with the glass plate, was quickly immersed in a coagulation bath containing distilled water for 30 min to stabilize their structure at room temperature (22 ± 1 °C). Before using them for experiments, the prepared biopolymeric membranes (CA/PEG/CHI-Ag) were washed with bi-distilled water. The biopolymeric membrane without CHI-Ag (CA/PEG) was prepared through a similar procedure.

**Figure 1:**
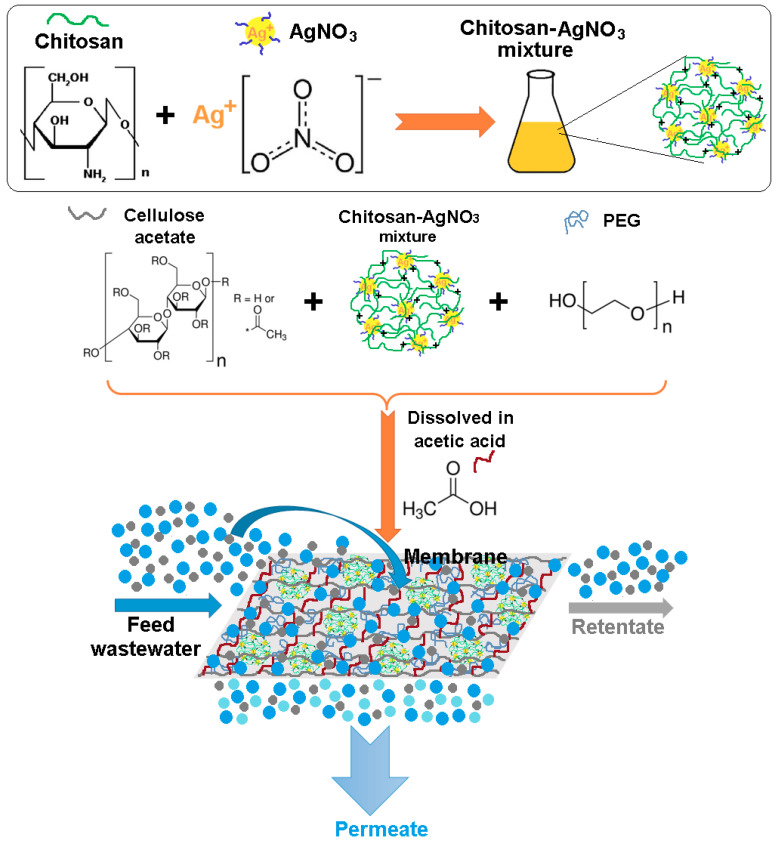
Schematic illustration mechanisms for the obtaining of the biopolymeric membrane for removal of metal ions by electrodialysis process.

The possible interconnections, interactions, intermolecular and cross-linked polymeric networks, which can occurred during the preparation of biopolymeric membrane for removal of metal ions by electrodialysis process, are schematic illustrated in Figure 1.

### 2.3. Characterization Techniques and Instrumentation

#### 2.3.1. Fourier Transforms Infrared Spectroscopy-Attenuated Total Reflection (FTIR-ATR)

Infrared spectroscopy (IR) study was performed on Perkin Elmer Spectrum 100 FTIR spectrophotometer (PerkinELMER, Ltd, London, United Kingdom) in the attenuated total reflection mode (ATR). The FTIR-ATR spectra between 4000–600 cm^−1^ were recorded by collecting 16 scans for each point, at a spectral resolution of 4 cm^−1^.

#### 2.3.2. Contact Angle and Surface Free Energy (SFE)

The wetting behavior of the obtained membranes was evaluated by contact angle measurements using a Contact Angle Meter (Kyowa Surface Chemistry Co., Ltd., Tokyo, Japan), KSV instruments CAM 100 equipment (KSV Instruments, Helsinki, Finland). For each membrane, the procedure was repeated three times at room temperature, with a constant volume of distilled water (10 µL). The static contact angle formed between membrane and different liquids drop (water, dimethyl sulfoxide, and glycerol) was measured, and surface free energy (SFE) was calculated. The contact angle value represents the average of 5 measurements ± Standard Deviation (SD).

#### 2.3.3. Water Uptake

Water uptake was determined by the gravimetric method [16]. The prepared polymeric membranes were cut into small pieces with a size of 15 mm × 15 mm and kept in a beaker with 25 mL of distilled water for 24 h at room temperature (22 °C). After that, the polymeric membranes were taken out, and the excess water was removed from the polymeric membranes surface using filter paper and weighed using an analytical balance (Kern PCB 350-3, precision 0.001 g) (m_wet_). The biopolymeric membranes were immediately placed in an oven (DNO20-MRC, MRC Ltd., Holon, Israel), dried at 50 °C for 3 h, and then were weighed (m_dry_). The water uptake percentage was calculated applying Equation (1):

(1) $$\mathrm{Water}\;\mathrm{uptake}\;(\%)=\frac{m_{wet}-m_{dry}}{m_{dry}}\cdot 100$$

#### 2.3.4. Thermal Gravimetric Analysis (TGA) and Differential Thermal Analysis (DSC)

Thermogravimetric analysis (TGA) and differential thermal analysis (DSC) of the prepared membranes were conducted using a Q5000IR system (TA Instruments, New Castle, DE, USA). Briefly, 4–14 mg of the sample was heated from 35 to 740 °C with a heating rate of 10 °C/min in platinum pans (100 µL), under nitrogen atmosphere (50 mL/min).

#### 2.3.5. Microscopy Studies

A digital optical microscope with high-resolution and full color 3.5” TFT LCD screen (Celestron LCD Digital Microscope II) was used to investigate the surface morphology of the prepared membranes without and with chitosan-silver ions.

The surface morphologies, top and cross-section view of the prepared biopolymeric membranes, were recorded using Scanning Electron Microscopy (SEM) (S-2600N, HITACHI). The biopolymeric membranes were cut into pieces of 0.5 × 0.5 cm and then covered with a thin gold layer. The experiments were performed in liquid nitrogen.

#### 2.3.6. Electrochemical Impedance Spectroscopy

**Figure 2:**
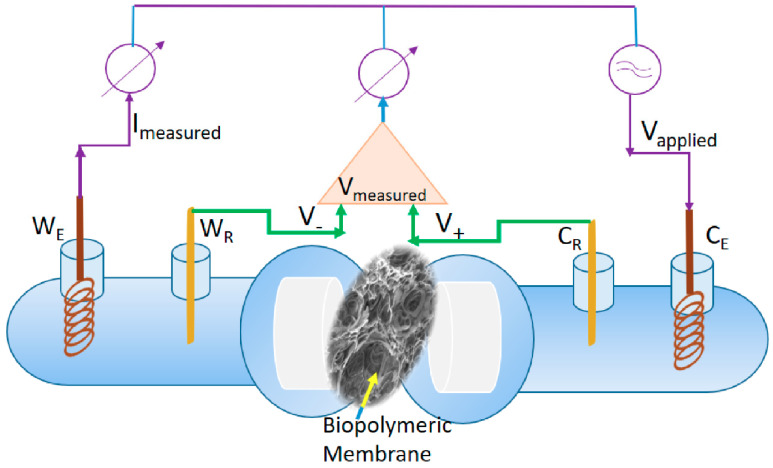
Scheme of the used impedance cell with four electrodes. W_E_—working electrode; W_R_—reference electrode associated with the W_E_; C_E_—counter electrode; C_R_—reference electrode associated with the C_E_; I_measured_ is the measured current; V_measured_ is the measured potential; V_applied_ is the applied potential.

The bulk resistance of the membranes was determined through the electrochemical impedance spectroscopy. The experimental set-up was performed as previously indicated in our studies [40,41,42] using a specific symmetrical four electrodes impedance cell (Figure 2) with two pairs of Ag/AgCl electrodes. One pair of electrodes will allow the current passing, and the other will measure the potential difference across the polymeric membrane.

All electrochemical impedance experiments have been carried out at an ambient temperature, in the presence of the ferrous electrolyte (10^−2^ mol/L). The electrical parameters of the membranes will be extracted from the equivalent circuits associated with the membranes.

A Parstat 2273 Electrochemical System (Princeton Applied Research University, USA) was used for all impedance measurements. The impedance cell made from Jena glass and presented in Figure 2 was manufactured at the University Politehnica of Bucharest facilities. The presented cell allowed the measurement of the voltage and the current at the membrane level using all four available channels of the electrochemical system. Figure 2 presents the four active electrodes–the working electrode (W_E_) and the counter electrode (C_E_) with their associated reference electrodes (W_R_, C_R_). The reference electrodes connected through an operational amplifier gave the measured potential (V_measured_). At the level of C_E_ is applied the selected voltage (V_applied_) and the total current is provided, while the W_E_ measures the current (I_measured_). The electrodes were made from 0.5 mm pure Ag wire (99.9%, Sigma Aldrich, Merck KGaA, Darmstadt, Germany), either as Ag rods or coiled AgCl coated Ag wire.

The area of the cut polymeric membranes, used in the impedance cell, was 0.785 cm^2^_,_ and the width 0.22 mm. The applied frequency range was from 1.0 Hz to 2.0 MHz, and the applied a.c. amplitude was 10 mV. For all experiments, the data integration was set at 5 cycles and 10 steps per decade.

The electrochemical impedance investigations allow the assessment of the main electrical parameters of the polymeric membranes studied, namely: resistance, capacitance, dielectric constant. The dielectric constant, known as well as relative electrical permittivity, is determined from the measured geometrical capacitance and the geometrical characteristics of the polymeric membranes: width and surface. The geometrical capacitance was calculated using both the Nyquist and Bode plots and was determined according to the Equation (2) [40,41,42]:

(2) $$C_{g}=\frac{l}{2\cdot \pi \cdot \gamma \cdot R_{\mathrm{ct}}}$$

where: C_g_ represents the capacitance (F); γ stands for the frequency at the maximum recorded on the Nyquist plot (Hz); R_ct_ is the charge transfer resistance of the membrane (Ω). The value of R_ct_ was determined using the Nyquist diagram as the difference between the intercept of the semicircle having the Z’ axis in the region of low frequencies and the intercept of the semicircle having real axis in the region of high frequencies. The accurate value of the frequency recorded for the maximum point was established from the Bode plot.

The relative electrical permittivity, which depends on the homogeneity of the surface, is given by Equation (3) [40,41,42]:

(3) $$C_{g}=\frac{\varepsilon_{0}\cdot \varepsilon_{r}\cdot A}{l}\;\to \;\varepsilon_{r}=\frac{C_{g}\cdot \varepsilon_{0}\cdot A}{l}$$

where: C_g_ represents the capacitance (F); ε_r_ is the relative electrical permittivity of the polymeric membrane; ε_0_ represents the electrical permittivity of the free space (8.854 × 10^−12^ F/m); A is the surface (1.53 ×10^−4^ m^2^), while l is the width (2.2 × 10^−4^ m) of the membrane.

The dielectric constant gives essential information on the charge transporting ability of the polymeric membranes. Conductivity, another significant electrical parameter, was calculated using the experimental data provided by the Nyquist diagram, according to the Equation (4) [40,41,42]:

(4) $$\sigma =\frac{l}{R_{ct}\cdot A}$$

where: σ is the electrical conductivity (Ω^−1^·m^−1^); R_ct_ is the charge transfer resistance (Ω); l is the thickness (m), and A is the surface of the membrane (m^2^). The distribution of both carrier concentration and mobility within a membrane will lead to the distribution of conductivity (σ).

### 2.4. Electrodialysis Equipment and Procedure

**Figure 3:**
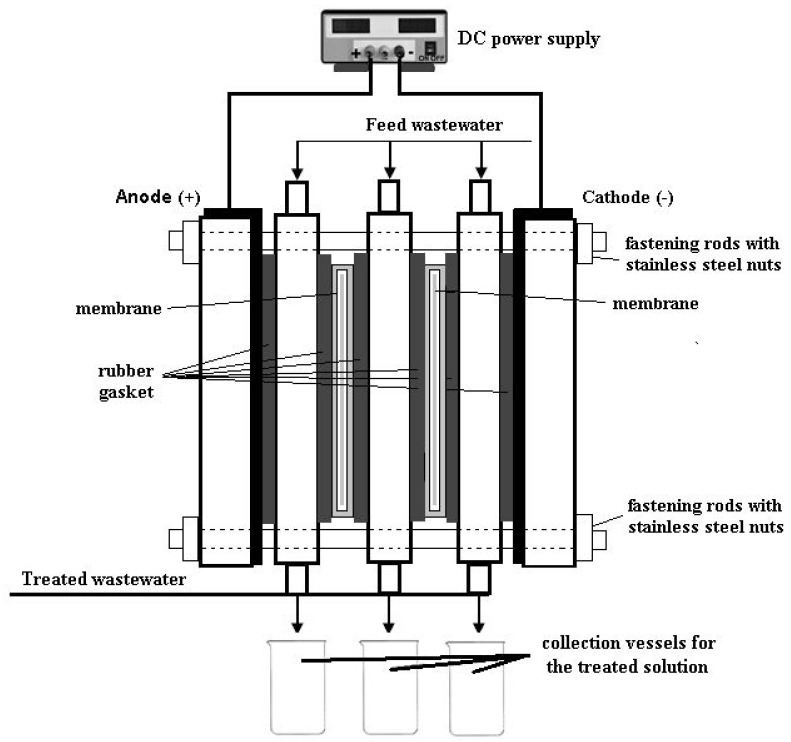
Schematic representation of the electrodialysis system.

Experiments were carried out in an electrodialysis system with three circular compartments made from high-density polyethylene with coextruded polypropylene exfoliating protective layer. The electrodialysis system was equipped with two parallel circular lead electrodes (purity 99.9%, anode, and cathode), placed at the ends of the electrodialysis cell. The obtained biopolymeric membranes were positioned between the compartments (Figure 3). The thickness of each compartment, with rubber gaskets (Yato YT-0687) on both sides of the compartment, was 11.6 mm. The effective surface area of each biopolymer membrane and each electrode, under investigation, was 14.3 cm^2^. The thickness of each electrode was 0.24 mm. The biopolymer membrane thickness was between 0.17 mm and 0.22 mm. The dimensions of electrodialysis cell, membrane, and electrodes thickness were performed with a Neiko 01407A electronic digital caliper stainless steel body with LCD screen (inch/fractions/millimeter conversion).

All experiments were accomplished with synthetic industrial wastewater prepared using ferrous sulfate heptahydrate, 10 mL of sulfuric acid 1 N, and deionized water to produce a wastewater feed of 100 ppm iron ions. The compartments were filled with a total volume of approximately 50 cm^3^ feed synthetical wastewater. The electrodialysis experiments were performed at room temperature (22 ± 1 °C) and operated under different constant voltages (5, 10, and 15 V) (galvanostatic operation mode) imposed at electrodes by a power source (AXIOMET AX-3005D) for 1 h (each experiment). In the electrodialysis system, the applied voltage produces the transportation of the cations (Fe^2+^) towards the cathode electrode and the anions (SO_4_^2−^) and hydroxyl ions (OH^-^) towards the anode electrode. Also, the migration of iron ions occurs across the membranes under the applied voltage at the electrodes.

All solutions, after each experiment, were filtered using the filter paper (Whatmann paper, 0.45 µm) to minimize the interference of fine particles in suspension with the analysis. The filtrates were analyzed for residual concentrations of iron ions using a UV-visible spectrophotometer Metertech SP-830 at a wavelength of 510 nm.

## 3. Results and Discussion

### 3.1. Membranes Characterization

#### 3.1.1. FTIR-ATR Spectroscopy Characterization of Membranes

**Figure 4:**
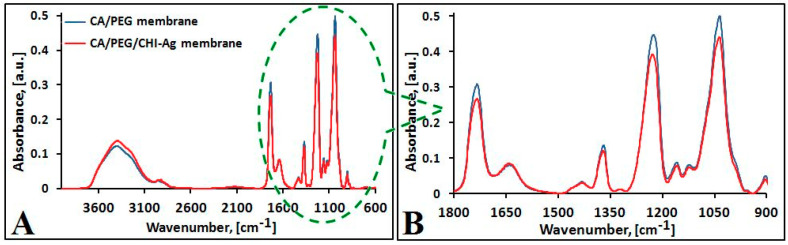
FTIR-ATR spectra of prepared membranes: CA/PEG (**A**) and CA/PEG/CHI-Ag (**B**).

To identify the specific absorption bands of each component and to establish the interaction between CA and PEG and CA, PEG, CHI, and silver (Ag) ions, infrared spectroscopy was used. The FTIR-ATR spectra of the obtained membranes are presented in Figure 4.

FTIR-ATR spectra of polymeric membranes, with and without chitosan (CHI)—silver (Ag) ions, show various absorption bands in 4000–600 cm^−1^ domain (Figure 4) [43]. The incorporation of CHI-Ag into the polymer matrix occurred successfully, as shown by the following bands:

- The stretching vibration band related to the—OH group (ν_O-H_) was observed at 3405 cm^−1^ for the polymeric membrane without chitosan-silver ions [44]. An intense absorption band was observed at 3395 cm^−1^ when CHI-Ag was added to polymer mixture, corresponding to ν_O-H_ overlap to the stretching vibration of N–H (ν_N-H_) from the free amino group (-NH_2_) at the C_2_ position of glucosamine from CHI. This absorption band registered by prominent peaks suggests the hydrogen bonds formed between components. Comparing the membranes’ spectra, it can be observed that the presence of CHI-Ag into the composition led to a more intense peak shifted to lower wavenumber (about ~10 cm^−1^).
- The aliphatic C-H stretching was registered at 2956 cm^−1^ and 2897 cm^−1^, respectively, in the polymeric membrane without chitosan-silver ions (CA/PEG/CHI-Ag membrane) [45].
- The same band corresponding to valence’s vibrations of C-H (ν_asC–H_) in –CH_2_– and in –CH_3_ (ν_s C–H_) appeared at 2956 cm^−1^, 2920 cm^−1^, and 2853 cm^−1^ in the case of the polymeric membrane enriched with chitosan-silver ions. The new peak at 2920 cm^−1^ and the shifting from 2897 cm^−1^ to 2853 cm^−1^ are due to the CHI-Ag incorporation in the mixture.
- The carbonyl group stretching vibration (ν_C=O_) specific to CA appeared in both membranes at 1733 cm^−1^ [46]. Also, the presence of this band suggested the formation of O–H· · ·C=O interaction between CA and PEG and CA, PEG and CHI, respectively [47].
- Vibrations of–OH groups from PEG and CHI are evidenced by the peak at around 1431 cm^−1^ corresponding to δ_CH2_ in CH_2_OH groups.
- The peaks at around 1370 cm^−1^ and 1320 cm^−1^, respectively, appeared in both membranes and are related to the vibrations of –OH and –CH groups in the pyranose ring [48].
- Between 1159 cm^−1^ and 1035 cm^−1^, the presence of distinct vibrational modes like –C–O–C (ν_C-O-C_ in glycosidic linkage) and C-OH (ν_C-O_ in primary OH group) are noticed.

Significant changes occurred in the FTIR-ATR spectrum of the polymeric membrane enriched with chitosan-silver ions, primarily related to the modification of the absorption band intensities. The shifting of some bands reveals the incorporation of CHI-Ag into the polymer mixture.

#### 3.1.2. Contact Angle and Surface Free Energy (SFE) Determination

Contact angle measurements investigated the effect of incorporation of CHI and Ag ions into the polymer mixture on the membrane’s hydrophilic/hydrophobic capacity. A membrane with high hydrophobicity could lead to the deposition of particles on the surface reducing the membrane efficiency and its lifetime. The contact angle values of obtained membranes using different liquids (water, dimethyl sulfoxide and glycerol) are presented in Table 1.

The values presented in Table 1 show that the higher contact angle value was registered for the CA/PEG membrane (101°), indicating its hydrophobic character. In the case of the CA/PEG/CHI-Ag membrane, the contact angle decreased (90.12°) when water was used as liquid, which demonstrates the effect of CHI-Ag presence on the decrease in polymeric membrane hydrophobicity. For CA/PEG membrane, a higher contact angle value was registered, compared with CA/PEG/CHI-Ag membrane, indicating that the amino group presence from chitosan led to a decrease in hydrophobic capacity. In this case, high values of the contact angle may suggest a decrease in membrane’s reliability in time [49]. The contact angle offers a certain versatility of the prepared polymeric membrane for biomedical applications due to the antibacterial potential effect of the Ag ions trapped into the polymer matrix [21,34,50].

Moreover, to evaluate the chemical affinity between the liquid and the solid support, the surface free energy (SFE) was assessed. Using the contact angle values against three liquids (Table 1) and Owens–Wendt’s theory based on the Fawkes method [44], disperse and polar fractions of the surface tension for liquids and SFE for solids were calculated (Equations (5) to (7)). The surface tension parameters of used fluids [51,52,53] are presented in Table 2, and the obtained results are shown in Table 3.

(5) $$\gamma_{L}\cdot \left(1+\cos\theta \right)=2\cdot \sqrt{\gamma_{L}^{d}\cdot \gamma_{S}^{d}}+2\cdot \sqrt{\gamma_{L}^{p}\cdot \gamma_{S}^{p}}$$

(6) $$\gamma_{S}=\gamma_{S}^{d}+\gamma_{S}^{p}$$

(7) $$\gamma_{S}=\gamma_{L}^{d}+\gamma_{L}^{P}$$

where: θ represents the contact angle; γ_S_ is the total surface energy of the solid; γ_L_ is the total surface tension/surface energy of the liquid; $\gamma_{L}^{d}$ and $\gamma_{L}^{p}$ are dispersive and polar components of the surface tension, while $\gamma_{S}^{d}$ and $\gamma_{S}^{p}$ are the dispersive and polar components of the solid surface energy [51,52,53].

A decrease in contact angle value (Table 1), as well as an increase in total surface energy (Table 2) and the value of the polar components for the CA/PEG/CHI-Ag membrane, was noticed. The hydrophobic character of the CA/PEG membrane decreased due to the incorporation of CHI-Ag, and the increase of polar components favored the increase in energy values (Table 3).

The mixing of PEG and CHI-Ag solution into a polymeric matrix allowed the obtaining of biopolymeric membranes where the silver ions were entrapped throughout the chitosan cross-linked polymeric networks via amino (–NH_2_) and hydroxyl (–OH) functional groups. Due to its water solubility, PEG has a good dispersion capacity in polymer matrix [27]. The amino groups from chitosan are involved as chelating agent for the removal of iron ions, and the Ag ion influenced dielectric permittivity and electric conductivity [26,49].

#### 3.1.3. Water Uptake

The percentage of water uptake values (calculated according to the Equation (1) [54]) for CA/PEG/CHI-Ag membrane was 66.67%, and for CA/PEG membrane was 64.17%. A small difference can be seen between the values of the percentage of water uptake (%) for the two membranes due to the small amount of CHI-Ag (0.5 mL) included in the polymer solution. However, the value of the water uptake percentage CA/PEG/CHI-Ag membrane was higher than that obtained for the CA/PEG membrane, due to the immobilization of chitosan in the polymer solution, which leads to some changes in the membranes. This result correlates well with the spectral data obtained by FTIR. The interaction between CA/PEG and CHI-Ag led to a more compact polymer structure with improved iron ions removal efficiency from wastewater by electrodialysis process. This fact could be due to the interactions with the hydroxyl and amino groups from chitosan on the surface. Also, the presence of Ag ion influenced the water uptake which is important for iron ion transport process [49,54].

#### 3.1.4. Thermal Gravimetric Analysis (TGA) and Differential Thermal Analysis (DSC) Characterization

**Figure 5:**
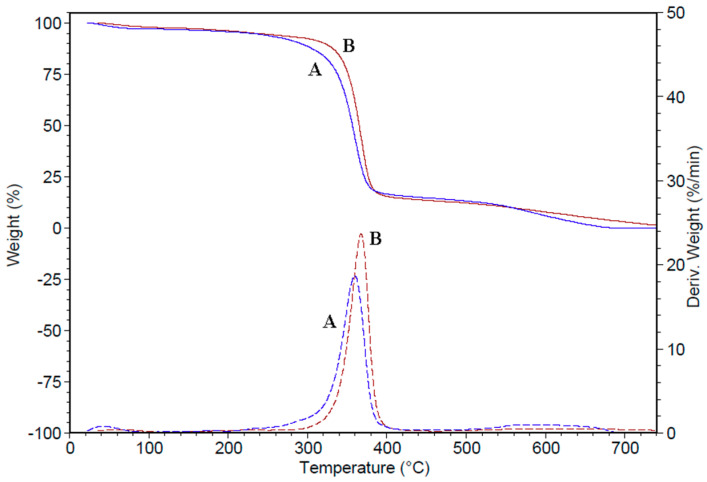
TGA-DSC curves of prepared membranes: CA/PEG (A) and CA/PEG/CHI-Ag (B).

TGA-DSC analysis of prepared membranes was performed from 35 to 740 °C in a nitrogen atmosphere (Figure 5).

The thermal properties of the prepared polymer membranes are different due to the presence of CHI-Ag in the polymer matrix (Figure 5 and Table 4). The first weight loss reduction appeared because the water contained in the sample evaporated [55]. The prepared polymer membranes show excellent thermal stability up to 350 °C, the maximum decomposition (T_max_) appearing at 367.5 °C in the case of the CHI-Ag membrane and at 359.6 °C for the polymer membrane without CHI-Ag. The degradation starts later for CA/PEG/CHI-Ag because of loose networks or moisture present due to the nanoparticle’s incorporation process, leading to porosity. The significant weight loss was found to be 81% for CA/PEG/CHI-Ag membrane, from 251.5 to 457.5 °C. Such weight loss could be assigned to the decomposition of cellulose acetate and chitosan. The last weight loss of 11.92% was observed between 457.5 and 740 °C and can be due to the decomposition of CA/PEG/CHI-Ag. The CA/PEG/CHI-Ag membrane has higher stability than CA/PEG membrane. The difference of the residual mass at 740 °C is due to the presence of silver nanoparticles in the membrane [21,50,56].

The T_max_ is higher for CA/PEG/CHI-Ag membrane in comparison with the CA/PEG membrane, which may result due to the incorporation of CHI-Ag in the polymer matrix (Table 4). The thermal stability of prepared polymeric membranes is affected by the dispersion of silver ions and chitosan in the polymer matrix, hydrogen bonding, and interaction of polymer chains and silver ions [50,56]. Also, the T_max_ increased for the membrane, which contains CHI-Ag, indicating that the Ag ions can modify the thermal stability of the CA/PEG/CHI-Ag membrane. This phenomenon can be ascribed to the fact that the overall crystallinity of the polymer is increased, leading to enhanced thermal properties.

The DSC was carried out under the continuous flow of dry nitrogen gas (50 mL/min) at 10 °C/min heating rate. Figure 5 illustrates the thermograms of CA/PEG and CA/PEG/CHI-Ag membranes, respectively. In Figure 5, the endothermic event appeared between 35–80 °C, corresponding to water evaporation. The CA/PEG/CHI-Ag membrane exhibits increased melting temperature due to the addition of CHI-Ag into the polymer matrix [50,56].

#### 3.1.5. Microscopic Analysis

**Figure 6:**
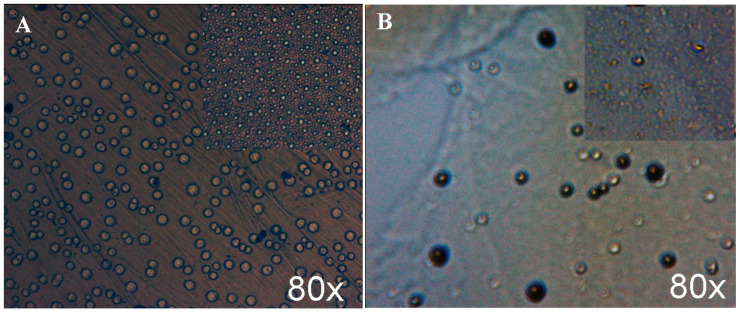
Optical microscope images for the surface morphology of prepared membranes: CA/PEG (**A**) and CA/PEG/CHI-Ag (**B**).

The surface morphology image at the macroscale (magnification 80×) for CA/PEG membrane shows a highly texturized and porous surface, with many opened pores, in comparison with the apparent homogeneous, compact, and flat surface for CA/PEG/CHI-Ag membrane (Figure 6). The optical microscopies images highlight that the CHI-Ag molecules were included in the obtained membrane (as spherical particles), and these decreased the rate of pores formation [34,50].

**Figure 7:**
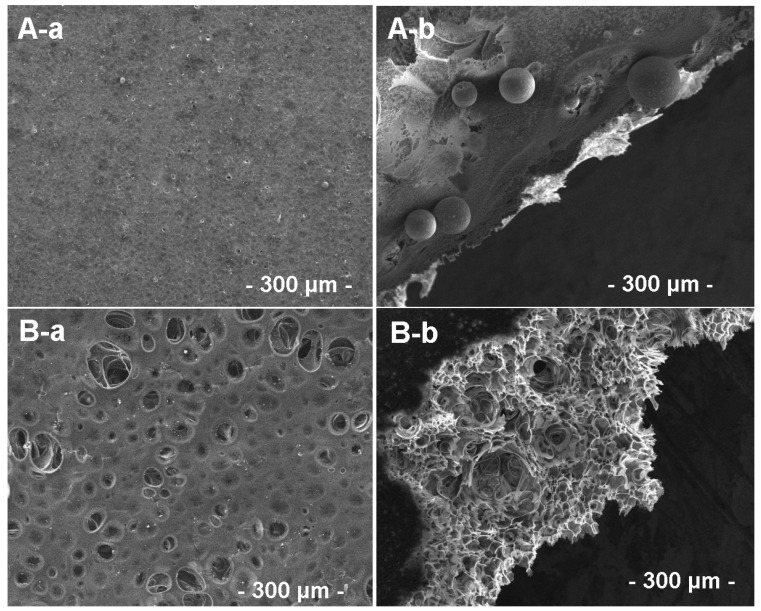
SEM images of prepared membranes: CA/PEG, top (**A-a**); CA/PEG, section (**A-b**); CA/PEG/CHI-Ag, top (**B-a**); CA/PEG/CHI-Ag, section (**B-b**).

The top and section surface morphology of the obtained polymeric membranes without and with chitosan (CHI)-silver (Ag) ions was examined by SEM (Figure 7).

SEM images (Figure 7B-a,B-b) indicate the presence of CHI-Ag ions into the prepared biopolymer. The CA/PEG/CHI-Ag membrane exhibited a dense and uniform plain microstructure with many small pores throughout the membrane. The SEM results illustrate that the pores formed have an average size between 6.12–12.64 µm for CA/PEG membranes (Figure 7A-a) and between 17.23–53.61 µm for CA/PEG/CHI-Ag membrane (Figure 7B-a). The significant decrease in the pore sizes could be due to the presence of the CHI-Ag ions in the polymer matrix and due to the involvement of further reduction as well as balancing the stabilization process by chitosan and PEG polymeric chains. It was observed that the CA/PEG/CHI-Ag membrane presents macro voids that are interconnected through the walls, which have small pores that could create a barrier to the transport of water and of metallic ions. The presence of pores and of macro voids can reduce the intrinsic membrane resistance for metallic ions transport [19]. The literature studies indicate that the silver particles presented in prepared biopolymeric membranes are within the useful range for essential antibacterial applications [21,50].

#### 3.1.6. Electrochemical Impedance Spectroscopy Tests

The a.c. impedance spectroscopy represents one of the essential techniques that allow the study of the electrical response of the membranes in terms of complex impedance, dielectric constant, or conductivity, among others [40,41,42]. The impedance configuration of the system under study could be explained as a sum of RC circuits in parallel or series combination, being presented in the complex form of Z’-resistive and Z’’-capacitive. The cell with four electrodes, and presented in Figure 2, allows the minimization of the impedance artifacts in the measurement of the biopolymeric membranes studied. During the standard impedance measurement of a polymer/composite material, placed between the two electrodes, the complex physical parameters (impedance, conductivity) are assessed from the current I_measured_ and the potential difference V_measured_ = V_+_ − V_−_ (see Figure 2). The classical experiments are based on the assumption that the potential dropped homogeneously across the polymeric film. However, this hypothesis is not valid in real systems when the studied materials are inhomogeneous, with ionic insertions, for instance, that generate important potential changes with significantly different profiles across the membranes. Such specificity of the system studied together with the necessity to avoid the measurement impedance artifacts, sustain the usage of the four electrodes cell for the electrochemical investigations. Much more, at low values for the frequency, below 100 Hz, the silver electrodes present higher values for the impedance compared to the silver-silver chloride electrodes, whose impedance decrease with frequency increasing.

As a consequence, the used silver-silver chloride C_R_ and W_R_ electrodes presented low impedance, comparable with the W_E_ and C_E_ impedances. During the experimental setup, it was taken into account that an applied voltage with high amplitude could cause a significant movement of ions inside/out the biopolymeric membrane that might lead to erroneous experimental data. Therefore, the used 0.01 V for voltage amplitude permitted a proper recording of the impedance spectra.

**Figure 8:**
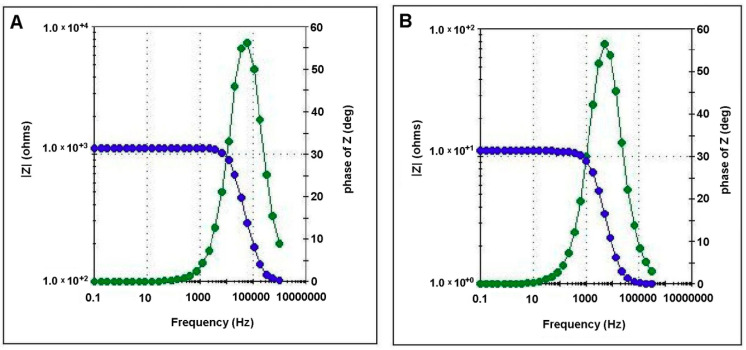
Bode diagrams for CA/PEG membrane (**A**) and CA/PEG/CHI-Ag membrane (**B**).

**Figure 9:**
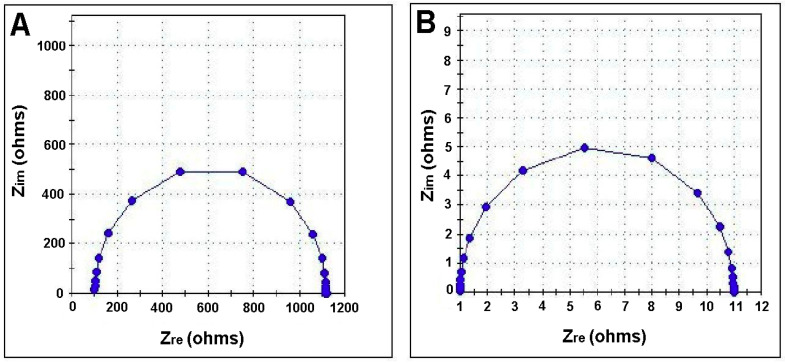
Nyquist diagrams for CA/PEG membrane (**A**) and CA/PEG/CHI-Ag membrane (**B**).

The electrochemical impedance spectra (Figure 8 and Figure 9) obtained for both biopolymeric membranes (without and with CHI-Ag), were characterized through fully resolved semicircles. The absence of any linear part shows that the exchange process at the level of the membranes occurs without diffusional control. For the prepared membranes, the observed single semicircle indicates a unique relaxation process.

The displacement of the semicircle on the diagram towards origin with the simultaneously decreasing in the semicircle size upon adding CHI-Ag in the membrane composition signifies that the membrane conductivity is not electronic, but ionic.

The set up in the four electrodes investigation cell (Figure 2) allowed us to assess the main electrical parameters with analytical interest for our study. The electrochemical behavior of the membranes was modeled using the equivalent circuits presented in Figure 9. For all samples studied, a single semicircle fit was recorded on the Nyquist diagram, indicating a single relaxation process. The *Z*′′ peak on the Nyquist plot shifted towards a higher frequency region when the membrane contained CHI-Ag. Such behavior suggests that electrical relaxation depends on membrane composition.

**Figure 10:**
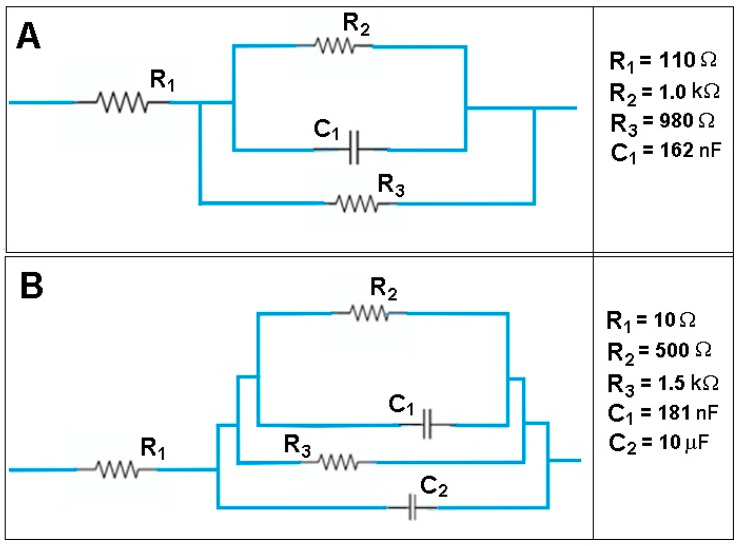
The equivalent circuits for CA/PEG membrane (**A**) and CA/PEG/CHI-Ag membrane (**B**).

As shown in Figure 10, the two equivalent circuits are similar. Compared to the membranes CA/PEG, in the equivalent circuit for the membrane with CHI-Ag, it was introduced in parallel to a capacitance, which is clearly due to the presence of CHI-Ag. The bulk resistances for the studied membranes varied quite significantly from 1160 Ω for the membrane CA/PEG to 10.9 Ω for the CA/PEG/CHI-Ag membrane. Therefore, it could be concluded that adding the chitosan-Ag into the membrane composition, the bulk resistance of the membrane was lowered by two orders of magnitude. The changes in the membrane resistance could be related to the variations in ion mobility within the composite membrane.

The diffusion coefficients of ions in membranes are strongly dependent upon the dielectric constant. A higher value for the membrane dielectric constant suggests a better ability of the membrane to hold a more substantial charge onto the membrane surface. The dielectric constant calculated according to the procedure presented in the experimental part (Equations (2) and (3)) was 10.41 for the CA/PEG membrane. In contrast, a value of 28.40 for the dielectric constant of the CA/PEG/CHI-Ag membrane was determined. Previously, literature data reported dielectric constant values for cellulose acetate membrane around 3.6 [57,58].

The linear amino polysaccharide compound that is chitosan (CHI) presents various functional groups, namely: –NH_2_, –C=O–NHR, or –OH. In complex polymeric matrix as cellulose acetate-PEG-CHI, the presence of silver particles influences the dielectric permittivity and electric conductivity that have a significant role in the ferrous ion transport mechanism.

The addition of CHI-Ag to the initial polymeric matrix cellulose acetate-PEG increases the conductivity of the final membranes. Such an increase of the conductivity values could be assigned to the possible movement of Ag charge carriers. Therefore, we might state that there are two routes for cation transport through the membrane: continuous narrow water channels and the bulk membrane hydrophobic phase. For the initial membrane, cellulose acetate-PEG, a value of 5.49 × 10^−4^ S/cm has been obtained for the electric conductivity. In other papers, for cellulose acetate-PEG membrane, values of the electrical conductivity between 2.12 × 10^−5^ S/cm and 9.5 × 10^−4^ S/cm [59] have been reported, but through another experimental procedure. Nevertheless, based on the impedance/diffusion cell used in the impedance investigations, the determined experimental values are realistic for the chemical species transportation.

The rapid movements of the polymer matrix segment, and the silver particles, generate a higher ionic electrical conductivity, as it is the case of the membrane with CHI-Ag when σ = 5.89 × 10^−2^ S/cm. The inclusion of an inert filler as silver in the polymeric matrix resulted in a higher ionic electrical conductivity.

The electrochemical impedance analysis, applied in this study, has the important advantage of an accurate assessment. The influence that the interaction between polymer matrix and the analytical aqueous solution has on the membrane’s charge transfer ability was quantified through the dielectric constant.

### 3.2. Performance Evaluation of Electrodialysis Cell

Based on preliminary data, which showed that a low applied voltage (<5 V) leads to a low treatment rate of iron ions, and a voltage over 15 V leads to cracking of polymeric membranes and increases energy consumption, there were chosen the values of applied voltages of 5, 10, and 15 V, respectively. Also, a concentrated synthetic industrial wastewater (over 100 ppm) was not studied due to the high energy consumption, damage, and clogging of membranes, which eventually increases the total cost. Moreover, after 1 h of treatment, it was observed that the energy consumption is higher and, also, the prepared membranes shows cracks. These effects lead to higher total costs, which are not an advantage. In this paper, we have included the best results obtained using the electrodialysis system with the biopolymeric membranes without and with chitosan (CHI)-silver (Ag).

The treatment rate of the iron (II) ions (T_R_ %) in the solutions was calculated by the Equation (8) [13,14,15,16,17]:

(8) $$T_{R}\;(\%)=\frac{C_{i}-C_{f}}{C_{i}}\cdot 100$$

where: C_i_ and C_f_, were the initial and final iron (II) ions concentrations (g/L).

The current efficiency (I_E_ %) was calculated based on the concentrations of solutions using Equation (9) [15,16,17]:

(9) $$I_{E}\;(\%)=\frac{z\cdot \left(C_{i}-C_{f}\right)\cdot V\cdot F}{N\cdot \bar{I}\cdot t}\cdot 100$$

where: I_E_ (%) is current efficiency; z is the valence of the iron ion; V is the total volume from all compartments (L); F is the Faraday constant (96,486 C/mol); N is the number of repeat cells; Ῑ is the average current intensity (A); t is the time of experience (s).

The energy consumption (W_c_, kWh/m^3^) of treatment by electrodialysis cell was calculated using the following Equation (10) [13,14,15,16,17]:

(10) $$W_{c}=\frac{1}{V\cdot 1000}\cdot \int_{0}^{t}U\cdot I\cdot dt$$

where: W_c_ is the energy consumption (kWh/m^3^); U is the applied voltage of the cell (V); I is the current intensity (A); t is the final of experience (h), and V is the total volume from all compartments (m^3^).

The calculated results for iron ions, after 1 h of electrodialysis cell, at different applied voltages, are indicated in Table 5.

The data presented in Table 5 highlight that the applied voltage that increased from 5 to 15 V causes an increase of the T_R_ from 15.86 to 33.71% for the polymeric membrane without chitosan-silver ions (CA/PEG membrane), and from 30.35 to 63.70% for the polymeric membrane enriched with chitosan-silver ions (CA/PEG/CHI-Ag membrane), after 1 h of treatment. The higher value of T_R_ resulted from a more intensive ionic migration with a higher voltage applied. At the end of each experiment, the formation of wet iron deposits on the cathode surface was observed. After drying, this deposit (iron powder) can be used in many different industries for different applications, such as brazing (sandpaper), chemicals, metallurgy (iron wires, iron cylinders, car parts, power tools), printing (toners, electrophotographic process), surface coating (iron plates, engine valves, steel rollers), dyes (if the iron powder is soaked in vinegar, a black, gray or brownish-red dye could be obtained, useful for craft makers and woodworkers) [16,60]. If, after the treatment of wastewater by the electrodialysis system, the water has the concentration allowed by the legislation imposed by the Environmental Protection Agency, then it can be released into the environment or it can be used as washing water, irrigation, or as rinsing water.

The increased treatment rate is due to the protons and hydroxyl ions (HO^-^) resulted from the water dissociation at the membrane surface [26,27]. Under the influence of electric fields, the protons move towards the cathode and hydroxyl ions (HO^-^) to the anode where they met with the Fe^2+^ through the membrane. Therefore, the hydroxide precipitate is formed onto the polymeric membrane surface [16]. In the literature, it was reported that under acidic conditions, the mobility of the adsorbate to the bio-sorbent surface is inhibited because the amino group (RNH_3_^+^) and the M^2+^ exhibit an electrostatic repulsion. The adsorption process of metal ions on chitosan is controlled by the transport of these species in solution [61].

Analyzing Table 5, it could be seen that the W_c_ increases when the applied voltage increases due to the amount of electrical intensity flowing between the electrodialysis cells. At 15 V, it was recorded a higher value of W_c_ (48.73 kWh/m^3^) for the polymeric membrane without chitosan-silver ions (CA/PEG membrane), compared to 37.30 kWh/m^3^ for polymeric membrane enriched with chitosan-silver (CA/PEG/CHI-Ag membrane). The presence of the iron ions causes a decrease in current efficiency for the polymeric membrane without chitosan-silver ions (CA/PEG membrane) and an increase in the energy consumption when the applied voltage increases. The T_R_ increased with the increasing applied voltage, but the water dissociation took place when the voltage increased to 15 V. This fact leads to decrease the current efficiency with increase of the applied voltage [17]. The I_E_ decreases as the iron ions concentration starts to increase in comparison with the concentration from the central compartment due to diffusion of these ions and osmotic water flow. At 15 V, the value of I_E_ (9.42%) was higher for the polymeric membrane enriched with chitosan-silver ions (CA/PEG/CHI-Ag membrane) compared to 7.71% for the polymeric membrane without chitosan-silver ions (CA/PEG membrane). This fact may be attributed to the competing effects of higher dissociation degree of water at the biodegradable polymer membrane level when the applied voltage was 15 V. After 1 h of electrodialysis, the total voltage and the resistance increased possible due to the depletion of iron ions in the dilute solution and due to the heating of the solution from electrodialysis cell. It had been reported in the literature that the constant voltage applied to the electrodes crosses the electrodialysis system. Due to the applied voltage as a function of the total resistance of the system, the electrodialysis system can be compared to a series of resistors, which includes the electrode rinsing solution resistance, the electrode reaction resistance, the membrane resistance, and finally, the dilute and concentrated solution resistances [17]. As iron ion transport is produced during the electrodialysis process, a dilute solution was flowing across the system (central compartment). It was observed that the resistance continuously increases, and the total resistance increases in the electrodialysis system. Due to this fact, the total voltage increases under galvanostatic control.

## 4. Conclusions

The results showed that the biopolymeric membrane enriched with the chitosan-silver ions can be successfully used for the removal of iron ions from synthetic industrial wastewater using a new electrodialysis system. The obtained data indicated that the efficiency increased due to the incorporation of CHI-Ag into the CA/PEG membrane. The highest value of the treatment rate of the iron ion (>60%) was obtained for biopolymeric membranes enriched with the chitosan-silver ions, at 15 V, after one hour of treatment.

FTIR-ATR study evidenced that hydrogen bonds’ interactions between the components in the mixtures occurred, and a complex of the polymeric matrix and metal ion was obtained. The TGA-DSC analysis for the obtained membranes showed excellent thermal stability (>350 °C), the maximum decomposition appearing at 367.5 °C for the biopolymeric membrane enriched with the chitosan-silver ions and at 359.6 °C for the biopolymeric membrane without chitosan-silver ions. The microscopic studies showed that the presence of CHI-Ag ions into the prepared biopolymeric membrane determined a decrease in the pore sizes.

The contact angle measurements evidenced different values for the prepared membranes with different compositions. The amino groups from chitosan presented in CA/PEG/CHI-Ag membrane composition have the key role in decreasing the hydrophobic capacity. Also, the hydrophobicity decreased with the increase of the polar components, favoring the increase in surface energy values.

The impedance spectroscopy studies allowed the assessment of the electrical conductivity of the prepared membranes. The value of this electrical parameter was improved from an initial 5.49 × 10^−4^ S/cm for the biopolymeric membrane without chitosan-silver ions to 5.89 × 10^−2^ S/cm for biopolymeric membrane enriched with the chitosan-silver ions. Such an increase in conductivity was assigned to the movement of the silver charge carriers or polymer matrix segments. The electrical characteristics of the biopolymeric membrane enriched with the chitosan-silver ions highlighted the suitability of the system for retaining iron ions from wastewaters.

The value of current efficiency was higher for the polymeric membrane enriched with chitosan-silver ions (9.42%) in comparison with the polymeric membrane without chitosan-silver ions (7.71%), and may be due to the competing effects of higher dissociation degree of water at the biodegradable polymer membrane level when the applied voltage was 15 V. The total voltage and the resistance increased was possible due to the depletion of iron ions in the dilute solution and due to the heating of the solution from electrodialysis cell.

The obtained biopolymeric membranes, due to their remarkable properties, could be used in different applications such as fuel cells, membrane processes (microfiltration, ultrafiltration, nanofiltration, reverse osmosis, ultrafiltration), and lately for thorough cleaning of clinical wastewaters.

## Figures and Tables

**Table 1:** Contact angle values of obtained polymeric membranes using different liquids.

Liquid	Contact Angle (°)	
	CA/PEG Membrane	CA/PEG/CHI-Ag Membrane
Water	101 ± 1.12	90.12 ± 0.62
Dimethyl sulfoxide	85 ± 0.72	60 ± 0.45
Glycerol	75 ± 0.68	74 ± 0.41

**Table 2:** Surface tension values of liquids used for contact angle measurements.

Liquid	Surface Energies [mN/m] Owens-Wendt		
	γ_L_	$\gamma_{L}^{d}$	$\gamma_{L}^{p}$
Water	72.8	21.8	51
Dimethyl sulfoxide	44	36	8
Glycerol	63.4	37	26.4

**Table 3:** Surface free energy (SFE) and its polar and dispersive components for obtained polymeric membranes.

Membrane	$\gamma_{S}^{P}$ mN m^−1^	$\gamma_{S}^{d}$ mN m^−1^	$\gamma_{S}=\gamma_{L}^{d}+\gamma_{L}^{P}$ mN m^−1^
CA/PEG	3.5280	14.7203	18.24828
CA/PEG/CHI-Ag	4.3786	21.6942	26.07273

**Table 4:** The weight loss (Wt. loss) and maximum decomposition temperature (T_max_) of CA/PEG and CA/PEG/CHI-Ag membranes.

Membrane	35–120 °C	120–202 °C		202–251.5 °C		251.5–457.5 °C		457.5–740 °C		Residue
	Wt. loss	Wt. Loss	T_max_	Wt. Loss	T_max_	Wt. Loss	T_max_	Wt. Loss	T_max_	740 °C
	%	%	°C	%	°C	%	°C	%	°C	%
CA/PEG	3.03	1.32	181.3	1.93	231.3	79.20	359.6	14.41	583.2	0.11
CA/PEG/CHI-Ag	2.36	1.56	194.5	1.76	229.8	81.00	367.5	11.92	625.9	1.40

**Table 5:** The values of T_R_ (%), I_E_ (%) and W_c_ (kWh/m^3^) at different applied voltages, after 1 h of electrodialysis experiment.

Applied Voltage (V)	CA/PEG Membrane			CA/PEG/CHI-Ag Membrane		
	T_R_ (%)	I_E_ (%)	W_c_ (kWh/m^3^)	T_R_ (%)	I_E_ (%)	W_c_ (kWh/m^3^)
5	15.86	9.57	6.99	30.35	27.33	4.10
10	29.43	8.07	29.88	41.09	25.56	10.13
15	33.71	7.71	48.73	63.70	9.42	37.30
